# New ARV drugs

**DOI:** 10.1186/1471-2334-14-S2-S2

**Published:** 2014-05-23

**Authors:** Roy Gulick

**Affiliations:** 1 Weill Cornell Medical College, Division of Infectious Diseases, New York, USA

## 

There are 28 approved antiretroviral drugs for the treatment of HIV infection; combinations of these drugs clearly change the natural history of HIV, dramatically decreasing HIV-related morbidity and mortality and promoting and prolonging healthy survival. Despite the benefits of HIV treatment, some antiretroviral regimens may be inconvenient, toxic, and/or have suboptimal antiretroviral activity, particularly against drug-resistant viral strains. Thus, newer compounds are needed that continue to improve convenience and tolerability, reduce toxicity, and improve antiretroviral activity, particularly against drug-resistant viruses. Additionally, newer drugs may better penetrate tissue reservoirs (e.g. genital tract, central nervous system) and/or exploit new viral or cellular targets with new mechanisms of action. In addition to the direct antiretroviral action, some investigational agents may target inflammation and/or immune responses. Finally, newer formulations of antiretroviral agents may allow additional options for one-pill, once-daily dosing or allow less frequent dosing than once-daily, perhaps as infrequently as once-monthly or once-quarterly parenteral dosing.

There are a number of investigational antiretroviral agents currently in development. These include newer antiretroviral agents in existing classes, including new nucleoside reverse transcriptase inhibitors, non-nucleoside reverse transcriptase inhibitors, integrase inhibitors, and entry inhibitors. Of those in the pipeline, a few compounds are in advanced stages of development: Tenofovir alafenamide fumarate (TAF) is a nucleoside analogue reverse transcriptase inhibitor currently in phase 3 that is potent and potentially less toxic than the current TDF formulation. Doravirine (formerly MK-1439) is a non-nucleoside analogue reverse transcriptase inhibitor currently in phase 2 that demonstrates activity against NNRTI-resistant viral strains. GSK ‘744 is an integrase inhibitor currently in phase 2, being developed in a long-acting preparation. In addition there are investigational drugs with new mechanisms of action in development: BMS-663,068, is a small molecule CD4 attachment inhibitor that completed phase 1 testing. Cenicriviroc is a novel CCR5/CCR2 antagonist currently in phase 3 that offers both antiretroviral activity and the potential for an anti-inflammatory effect (through CCR2 antagonism). A number of other compounds are in earlier stages of pre-clinical and clinical development.

The clinical use of these newer agents and formulations will depend on the results of clinical trials, and the timeline for development and availability. Continued progress in HIV drug development will allow improvements in the clinical care of patients living with HIV infection.

